# Integrating environmental and satellite data to estimate county-level cotton yield in Xinjiang Province

**DOI:** 10.3389/fpls.2022.1048479

**Published:** 2023-01-18

**Authors:** Ping Lang, Lifu Zhang, Changping Huang, Jiahua Chen, Xiaoyan Kang, Ze Zhang, Qingxi Tong

**Affiliations:** ^1^ State Key Laboratory of Remote Sensing Science, Aerospace Information Research Institute, Chinese Academy of Sciences, Beijing, China; ^2^ University of Chinese Academy of Sciences, Beijing, China; ^3^ Xinjiang Production and Construction Crops Oasis Eco-Agriculture Key Laboratory, College of Agriculture, Shihezi University, Shihezi, China

**Keywords:** remote sensing, climate variables, GEE, deep learning, yield estimation, cotton

## Abstract

Accurate and timely estimation of cotton yield over large areas is essential for precision agriculture, facilitating the operation of commodity markets and guiding agronomic management practices. Remote sensing (RS) and crop models are effective means to predict cotton yield in the field. The satellite vegetation indices (VIs) can describe crop yield variations over large areas but can’t take the exact environmental impact into consideration. Climate variables (CVs), the result of the influence of spatial heterogeneity in large regions, can provide environmental information for better estimation of cotton yield. In this study, the most important VIs and CVs for estimating county-level cotton yield across Xinjiang Province were screened out. We found that the VIs of canopy structure and chlorophyll contents, and the CVs of moisture, were the most significant factors for cotton growth. For yield estimation, we utilized four approaches: least absolute shrinkage and selection operator regression (LASSO), support vector regression (SVR), random forest regression (RFR) and long short-term memory (LSTM). Due to its ability to capture temporal features over the long term, LSTM performed best, with an R^2^ of 0.76, root mean square error (RMSE) of 150 kg/ha and relative RMSE (rRMSE) of 8.67%; moreover, an additional 10% of the variance could be explained by adding CVs to the VIs. For the within-season yield estimation using LSTM, predictions made 2 months before harvest were the most accurate (R^2^ = 0.65, RMSE = 220 kg/ha, rRMSE = 15.97%). Our study demonstrated the feasibility of yield estimation and early prediction at the county level over large cotton cultivation areas by integrating satellite and environmental data.

## Introduction

Cotton is an important cash crop used in fabrics, cloth, and oil. According to the International Cotton Advisory Committee (ICAC), China is the largest cotton consumer and second largest cotton producer in the world, and Xinjiang Province accounts for > 80% of the total cotton production of China. Precise estimation of the cotton yield of Xinjiang Province could inform Chinese and international policy decisions, and promote stable operation of agricultural commodity markets. Besides different genotype and management practices, extreme weather (e.g. droughts, floods, and high temperatures) also makes crop yield vary from year to year (Bauer et al., 2015). To prevent losses, it is necessary to measure the cotton yield in an accurate and timely manner for effective agronomic management practices (Xu et al., 2021a; Li et al., 2022b).

Satellite remote sensing (RS) is widely applied in agricultural research. Vegetation indices (VIs) calculated from satellite data are the most common means of predicting crop yield (Bian et al., 2022). VIs can describe such biotic features as the canopy structure, chlorophyll, and nitrogen content of crops and different indices indicate different features. The Normalized Difference Vegetation Index (NDVI), Enhanced Vegetation Index (EVI) and Near-Infrared Reflectance of Vegetation (NIRv) have been used to explain variation in wheat, corn, rice and soybean yields (Johnson, 2014; Meng et al., 2017; Fan et al., 2021). However, it is still not clear which RS VIs are optimal for predicting cotton yield and which biotic features are most relevant to the yield. Additionally, genotype (G), environment (E) and management (M), namely biotic and abiotic conditions have the greatest influence on crop growth and production (Jones et al., 2003; Tao et al., 2009). VIs alone have limited ability to estimate yield. Therefore, Climate variables (CVs) have also been applied by taking abiotic features into consideration at the same time. Temperature and precipitation are the most influential abiotic factors in crop breeding (Mathieu and Aires, 2018; Kang et al., 2020). However, their predictive power varies among regions. Precipitation does not precisely reflect the moisture available for plants between the sowing and mellowing stages. Since except for inevitable algorithm error in estimating precipitation value, the processes of crop growth are complicated. Water evaporation of leaves, and irrigation and drainage management practices, also affect moisture (Folberth et al., 2016; Chen et al., 2018). Vapor pressure (vap), vapor pressure deficit (vpd), reference evapotranspiration (pet), land surface temperature (LST), and the soil moisture resulting from the interaction of liquid and solar radiation in soil and vegetation have been used in analyses of the effects of climate changes on crop production (Rigden et al., 2020). Recent researches have shown that each satellite and climate index has advantages and disadvantages for predicting yield that depend on the diversity of the terrain and topography, spatial distribution of crops, and phenology (Tao et al., 2009; Kern et al., 2018). Therefore, it is necessary to investigate the relationships between VIs and environmental stress in the context of cotton crops over a large area.

The main approaches to crop yield estimation are crop models and regression methods. Biophysical models provide mathematical descriptions of crop growth and development in terms of radiation, photosynthetic production, respiration, transpiration, dry matter generation, and distribution (Sinclair and Seligman, 1996; Dorigo et al., 2007; Kheir et al., 2022). Process-based crop models consider all the G×E×M factors and their interactions. These models use daily crop type, soil, meteorology and field management data as input (Sinclair and Seligman, 1996; Keating et al., 2003; de Wit et al., 2019). However, the use of these high-quality inputs throughout the breeding and reproductive period is computationally intense (Tao et al., 2018). Although crop models for monitoring and predicting yield at a single location, or at the field scale, have made great progress, application to the regional scale is difficult due to the intricate data collection and huge calculation costs (Curnel et al., 2011; Wu et al., 2021). Statistical regression models are powerful tools applicable to large scales. They use fewer parameters and simpler inputs than the crop models, and are less computationally intense. Regression methods for yield prediction typically use optical satellite data instead of daily inputs over the entire growth stage. Moreover, they perform better than process-based crop models when there is a sufficient amount of training data (Liu et al., 2012). However, conventional linear regression methods have difficulty capturing the sophisticated relationships between various features, and may oversimplify the nonlinear relationships. Machine learning (ML) and deep learning (DL) algorithms can overcome the drawbacks of traditional statistical-based models. They disentangle the complicated relationships among input and target variables by fully training the model before practical application (LeCun et al., 2015; Ashapure et al., 2020). As well as having lower computational costs than biophysical models, DL and ML models can also assess the yield of numerous crops with greater accuracy and less error than linear regression approaches. DL methods have made particularly significant progress. They routinely involve hidden layers that abstract non-linear features to another dimensional space for linear partition as a black-box, thus simplifying the relationships among various inputs and outputs (LeCun et al., 2015; Chu and Yu, 2020). At the same time, these non-linear models are usually complex and difficult to interpret, highly dependent on data volume and need test sets to avoid overfitting. Nevertheless, ML and DL methods show excellent performance in terms of capturing the spatiotemporal variation of input data (van Klompenburg et al., 2020; Xu et al., 2021b). Recent studies have demonstrated the superiority of ML and DL methods for crop yield prediction. Support vector regression (SVR), random forest regression (RFR), convolution neural networks (CNNs), and long short-term memory networks (LSTM) have successfully estimated the yield of various crop types, considering the effects of climate change at the pixel or county scale (Gopal and Bhargavi, 2019; Sun et al., 2019; Khaki et al., 2020). To enhance ML and DL methods, the ensemble Bayesian model averaging (EBMA) and You Look Only Once version 5 (YOLOv5) which are improved models also applied (Wang et al., 2022; Fei et al., 2023). Furthermore, deep learning adaptive crop model (DACM) is proposed considering the spatial heterogeneity of large areas for yield estimation (Zhu et al., 2022). However, the basic ML and DL methods for cotton yield estimation are not sufficiently advanced for direct application to production and practice, especially in Xinjiang Province, China.

Regression models, including those based on ML and DL, require various parameters that are closely related to crop growth to narrow the gap between actual and potential (i.e. predicted) yield. The application of comprehensive RS and environmental data has improved crop yield predictions because different datasets contain diverse information on crop growth and development (Kamir et al., 2020; Zhang et al., 2020). For example, the green chlorophyll vegetation index (GCVI) combined with LST and other climatic indices explained about 70% of the variance in maize yield across China, with the LSTM showing the best performance (Zhang et al., 2021). Various environmental data for single and double rice systems have been integrated with the NDVI and EVI to predict rice yield in China (Cao et al., 2021). NDVI, Maximum temperatures and accumulated rainfall data were used to monitor Australian wheat yield (Kamir et al., 2020). Considering climate or weather conditions of crops within season, prediction of corn and wheat have been reached (Johnson, 2014; Jin et al., 2022). While for cotton yield estimation, most of the studies are limited to the field scale by means of remote sensing (Ashapure et al., 2020; Meng et al., 2021; Wang et al., 2021). These study areas are often dedicated to cotton fields in small areas. When we expand the study regions, spatial heterogeneity must be considered. Therefore, it is difficult to apply the methods and processes of the field scale over a large area. However, the optimum VIs and CVs for cotton yield estimation remain unclear, and the ability of ML and DL methods to predict early cotton yield also needs to be further explored.

**Figure 1:**
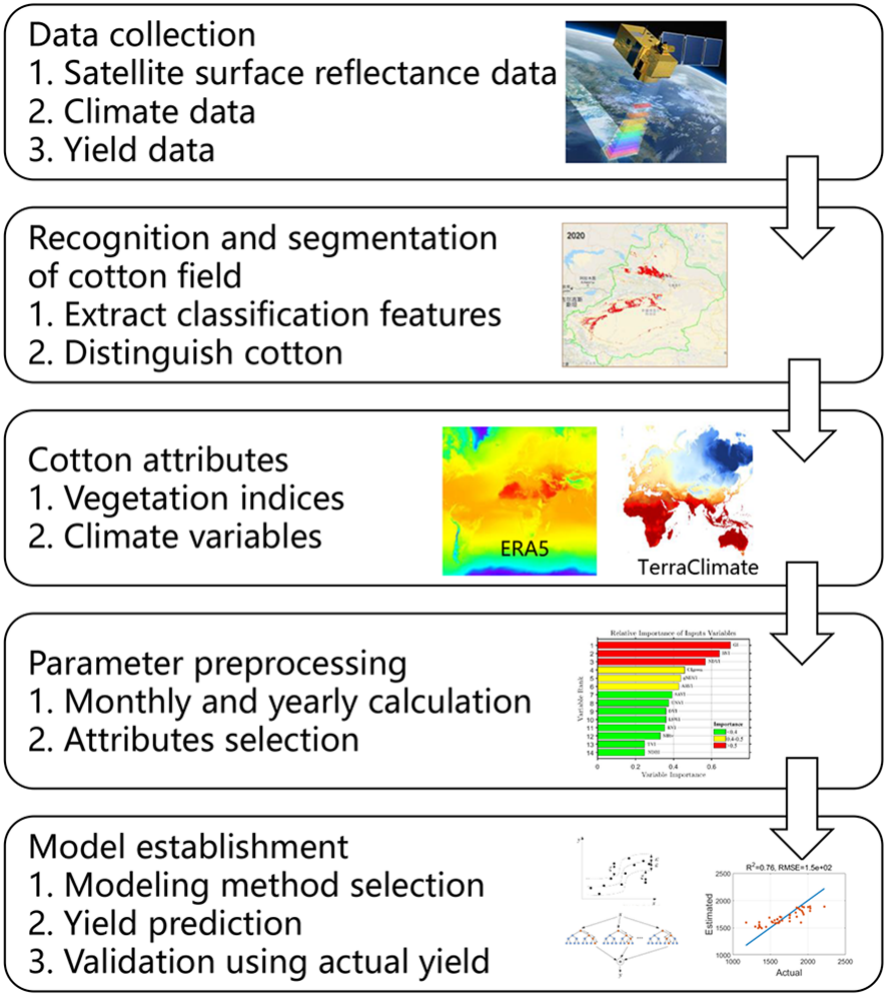
Workflow of county-level cotton yield prediction in this study.

Here, we used satellite data and environmental parameters to build regression models for accurate prediction of cotton yield from 2012 to 2019 at the county level in Xinjiang Province. Based on the extracted cotton field, we calculated VIs and CVs to screen out the best ones for model establishment. We used one linear (least absolute shrinkage and selection operator, LASSO), two ML (SVR and RFR), and one DL (LSTM) model. Our overall workflow is shown in **Figure 1**. We sought answers to three questions: (1) which VIs and CVs can most precisely describe the county-level cotton yield in Xinjiang Province? (2) which regression model best simulates cotton yield over a large area? (3) how long before harvest could the yield be predicted?

## Materials and methods

### Study region and cotton yield

**Figure 2:**
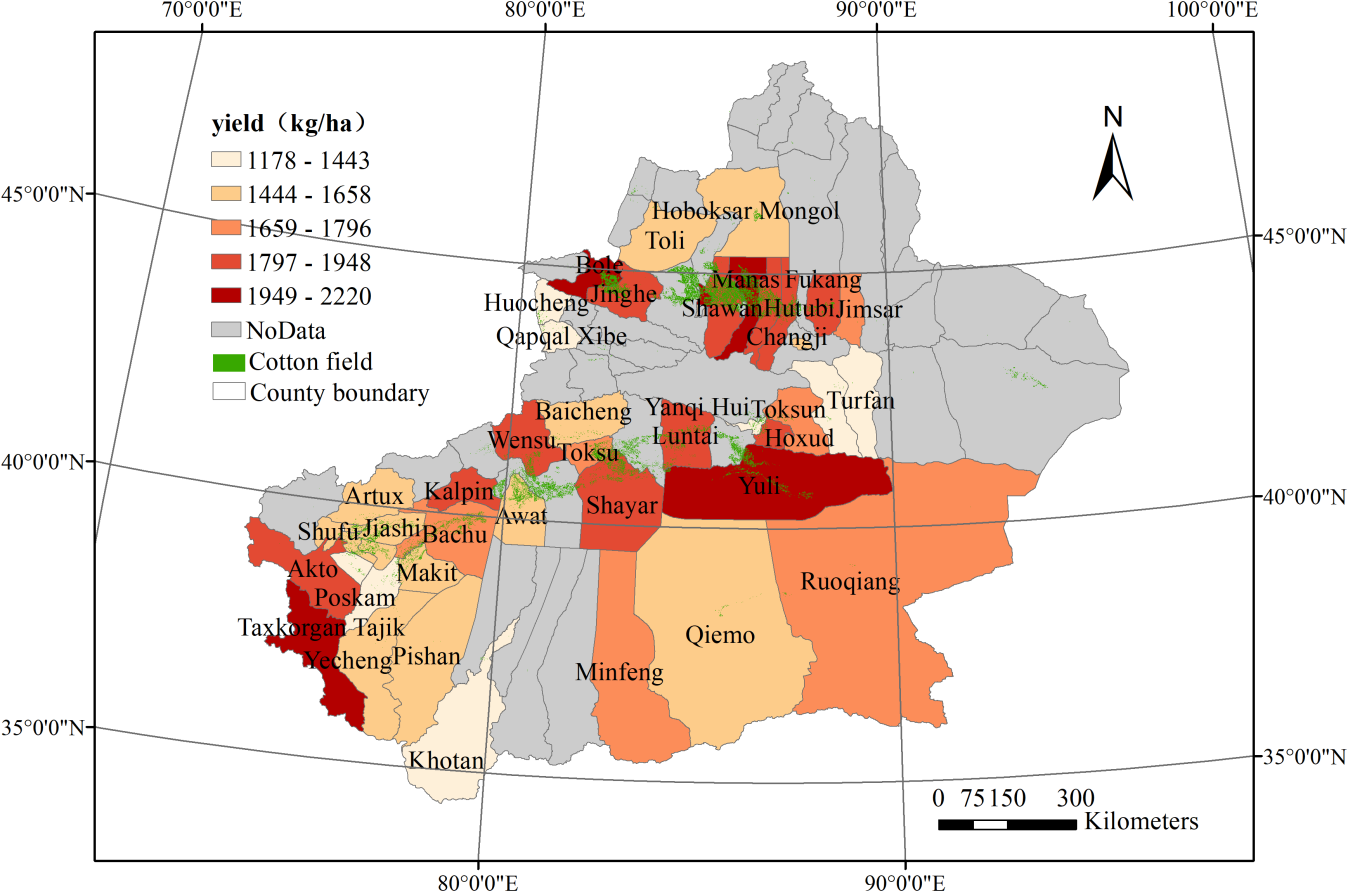
The study areas, cotton cultivation area and counties with recorded yield in Xinjiang Province in 2019. yields are from the Statistical Yearbook.

This study attempted to estimate cotton yields in Xinjiang Province (**Figure 2**), which produces more than 85% of the cotton grown in China. The study area, between 34°22′N-49°10′N and 73°40′E-96°23′E, covers approximately 166 million hectares. Xinjiang is among the districts in China most susceptible to climate change, as it spans the mid-temperate, south-temperate, and plateau climatic zones from north to south, with average daily air temperatures ranging from –28°C to 41°C and annual precipitation of about 150 mm. In Xinjiang, cotton is commonly planted in spring (April) and harvested in autumn (September–October; mostly in September). Therefore, we define the cotton growing season as the period from April to September.

County cotton yields (in kg/ha) from 2012 to 2019 were obtained from the agricultural statistical yearbook (https://www.yearbookchina.com). To reduce uncertainty, a preliminary quality check was used to identify and filter outliers, i.e. data points that were more than two standard deviations above or below the mean. Because of the special administrative structure of Xinjiang, the yield data did not cover the entire province. We selected counties with available cotton yield as yield records. In total, 355 yield records were used to define the study area (**Figure 2**).

### Satellite remote sensing and environmental data

Surface reflectance (SR) images of the cotton cultivation areas for 2012–2019 were acquired from MODIS (MOD09A1) and Sentinel-2 (L2A), and radiometrically calibrated and atmospherically corrected within the Google Earth Engine (GEE). After removing images with > 10% clouds, we masked the clouds in the remaining valid images using cloud-free bands. Based on these pre-processed images, 14 satellite VIs, including the NDVI, EVI, and Universal Normalized Vegetation Index (UNVI), were computed for yield prediction (**Table 1**). Annual and monthly averages of the MOD09A1 VIs were produced for 2012–2019, while only monthly averages for 2019 were produced for the Sentinel-2 L2A VIs. The annual values obtained by averaging the monthly means from April to September were used to predict cotton yield in 2012-2019. The monthly values were used to predict the yield before the cotton harvest and to explore the temporal pattern of cotton growth. To validate the feasibility of the MODIS dataset for estimating, the Sentinel-2 data of 2019 were used.

**Table 1:** Vegetation indices (VI) and their calculations.

Vegetation Index	Formulation	Reference
Normalized Difference Vegetation Index (NDVI)	$NDVI=\frac{Nir-Red}{Nir+Red}$	*(* Rouse, 1974 *)*
Enhanced Vegetation Index (EVI)	$EVI=\frac{2.5\times \left(Nir-Red\right)}{Nir+6\times Red-7.5\times Blue+1}$	*(* Huete et al., 2002 *)*
Green NDVI (gNDVI)	$gNDVI=\frac{Nir-Green}{Nir+Green}$	*(* Kaufman and Merzlyak, 1996 *)*
Triangular Chlorophyll Absorption Ratio Index (TVI)	*TVI*=60×*Nir*−*Green*−100×(*Red*−*Green*)	*(* Broge and Leblanc, 2001 *)*
Land Surface Water Index (LSWI)	$LSWI=\frac{Nir-Swir1}{Nir+Swir1}$	*(* Tucker, 1979 *)*
Green Index (GI)	$GI=\frac{Green}{Red}$	*(* Gitelson et al., 2003a *)*
Near-Infrared Reflectance of Vegetation (NIRv)	$NIR_{V}=Nir\times \frac{Nir-Red}{Nir+Red}$	*(* Badgley et al., 2017 *)*
Ratio Vegetation Index (RVI)	$RVI=\frac{Nir}{Red}$	*(Jordan, 1969)*
Difference Vegetation Index (DVI)	*DVI*=*Nir*−*Red*	*(* Tucker, 1979 *)*
Normalized Difference Built-up Index (NDBI)	$NDBI=\frac{Swir1-Nir}{Swir1+Nir}$	*(* Zha et al., 2003 *)*
Soil-Adjusted Vegetation Index (SAVI)	$SAVI=\frac{1.5\times \left(Nir-Red\right)}{Nir+0.5+Red}$	*(* Huete, 1988 *)*
Atmospherically Resistant Vegetation Index (ARVI)	$ARVI=\frac{Nir-\left(2\times Red-Blue\right)}{Nir+\left(2\times Red-Blue\right)}$	*(* Kaufman and Tanre, 1992 *)*
Green Chlorophyll Index (CIgreen)	$CI_{green}=\frac{Nir}{Green}-1$	*(* Gitelson et al., 2003b *)*
Universal Normalized Vegetation Index (UNVI)	$UNVI=\frac{C_{v}-0.1\times C_{s}-C_{4}}{C_{v}+C_{v}+C_{s}}$	*(* Zhang et al., 2019 *)*

Since precipitation, temperature, and soil all play important roles in plant growth, they are widely used for estimating crop yield (Kamir et al., 2020; Schwalbert et al., 2020; Gomez et al., 2021). We collected historical Climate Hazards Group Infrared Precipitation with Station data (CHIRPS) for daily precipitation (pre), ERA5 monthly temperature data [including maximum (Tmax), minimum (Tmin), and mean (Tmean) values], and TerraClimate data for monthly actual evapotranspiration (aet), climate water deficit (def), the palmer drought severity index (pdsi), precipitation accumulation (pr), soil moisture (soil), vapor pressure (vap), vapor pressure deficit (vpd) and reference evapotranspiration (pet) as climate parameters for the yield prediction models (**Table 2**). Yearly and monthly average values of the CVs were produced for 2012–2019. The same with VIs, the yearly and monthly values were for cotton yield estimation and prediction, respectively.

**Table 2:** Summary of the dataset used in this study.

Category	Variables	Spatial Resolution	Temporal Resolution	Time Coverage	Source
Crop yield	Cotton yield	Statistical division	Yearly	2012-2019	https://www.yearbookchina.com
Satellite data	MODIS VIs	500 m	8-day	2012-2019	https://lpdaac.usgs.gov https://doi.org/10.5067/MODIS/MOD09A1.006
	Sentinel-2 VIs	20 m	5-day	2019	https://sentinel.esa.int
Climate data	Pre	0.05°	Daily	2012-2019	CHIRPS (Funk et al., 2015)
	Tmax, Tmin, Tmean	10 km	Monthly	2012-2019	ERA5 (Horanyi, 2017; Hersbach et al., 2020)
	aet, def, pdsi, pet, pr, soil, vap, vpd	4 km	Monthly	2012-2019	TerraClimate (Abatzoglou et al., 2018)

### Cotton cultivation area

The cotton maps used to mask satellite and climate parameters during 2012–2019 were from our previous work. Based on high-spatial-resolution time series images that integrated Sentinel-2 and Landsat 8 satellite data, we explored the effects of image synthesis, the spectral index, and spatial texture on cotton identification accuracy, while also considering agricultural zoning. We applied the LSWI to a 10-day composite period analysis according to the farming division in Xinjiang, with texture features added at days 100, 200, and 260 to distinguish cotton from maize, wheat, and other main crops, and finally drew a spatial distribution map of cotton in Xinjiang in 2020. The map was verified with 5061 field samples obtained from ground surveys, with 3082, 466, 154, 341, and 1018 samples for cotton, maize, wheat, other crops, and non-farm land, respectively. The overall accuracy (OA) of cotton identification reached 0.8851, with a kappa coefficient of 0.8294, user precision of 0.9246, and producer precision of 0.9677. The specific spatial distribution of cotton cultivation areas shows in **Figure 2**

### Assessment of variable importance

To identify the most important yield predictors and discard unimportant variables, the relative importance of each input variable was calculated using the Boruta algorithm. It is essentially the same as the Random Forest Importance. They both were originated from the Random Forest but expressed in slightly different forms. The Boruta algorithm is a wrapper built around the random forest classification algorithm implemented in the R package randomForest in 2010 (Liaw and Wiener, 2002; Kursa and Rudnicki, 2010). It has also been introduced into Python, and the current Boruta version of Python is BorutaPy (https://github.com/scikit-learn-contrib/boruta_py). Boruta can iteratively remove less important features while running RFR. Based on the original feature, a shadow feature is derived *via* a shuffling process that extends the feature matrix. Then, the *z*-score is computed and the maximum value is used as the threshold. During each random forest run, original features with importance values exceeding the threshold are marked as important, while those with importance values below the threshold are marked as unimportant. In subsequent runs, the important features are included and unimportant ones are removed. When every original feature is marked as important or unimportant, or the random forest runs reach a previously defined limit, the algorithm ends.

### Prediction models

We first normalized the input variables using the *z*-score method, and then built regression models to determine their impact on yield. Four regression models were used to estimate cotton yield at the county level, i.e. a LASSO linear regression model, two ML models (SVR and RFR), and a DL model (LSTM), and their performances were compared. Due to insufficient valid data in the yearbook, 10-fold cross-validation, which can make full use of limited data, was applied. In the 10-fold cross-validation process, the dataset is evenly divided into 10 copies and each sample is labelled from 1-10. The cross-validation was repeated 10 times, once for each label as a test. During each run, the data with the same label are deemed as testing sets while the others are for training. For each prediction model, the averaged R^2^, root mean square error (RMSE) and relative RMSE (rRMSE) of 10 runs were used in the training and testing datasets for evaluating the performance. For within-season prediction result, the averaged R^2^, RMSE, rRMSE of 10 testing datasets were used.


(1) $$R^{2}=1-\frac{\sum_{i=1}^{n}{\left(y_{act,i}-y_{pre,i}\right)}^{2}}{\sum_{i=1}^{n}{\left(y_{act,i}-y_{ave}\right)}^{2}}\;$$

(2) $$RMSE=\;\sqrt{\frac{1}{n}\sum_{i=1}^{n}{\left(y_{act,i}-y_{pre,i}\right)}^{2}}$$

(3) $$rRMSE=\;\frac{RMSE}{y_{ave}}\;$$

where *y*_*act*_  is the actual true yield,*y*_*pre*_ is the model predictive yield,*y*_*ave*_ is the average*y*_*act*_ value, and*n* is the sample size. Details of the four models follow: LASSO regression is a shrinkage method characterized by variable selection and regularization that fits a generalized linear model (Tibshirani, 2011). The loss function can reduce the weight of input features to zero, which helps avoid overfitting. The LASSO uses the L1-regularization method to minimize the weight coefficient *ω* in the cost function [equation (4)]. The L1-penalty is the absolute value, which can’t get derivation directly. Therefore, the gradient descent method is used to approach the optimal solution gradually by iteratively updating the values of the weight coefficients along one of the coordinate axes. We ran the LASSO model from the *linear_model* package and let the parameter *alpha* to be optimized through the *GridSearchCV* function from the *sklearn* package in Python 3.8.


(4) $$Cost\left(\omega \right)=\sum_{i=1}^{N}{\left(y_{i}-\omega^{T}x_{i}\right)}^{2}+\lambda \omega$$

where *y*_*i*_  is the response value, *x*_*i*_  is the standardized predictors,*λ*  is the penalty coefficient,*ω* is the vector of weight coefficient, and*N* is the sample size.

SVR is a variant of a support vector machine that uses kernels to map input data in higher dimensional feature space, such that we can identify relationships between input and output variables (Drucker et al., 1996; Hsu and Lin, 2002). SVR uses hyperplanes that can minimize the error arising from training samples and make all data have the shortest distance from the plane [equation (5)]. This is a convex quadratic programming problem that can be solved by the Lagrange method. Of the various kernel functions, we used the radial basis function (RBF) instead of linear, sigmoid, or polynomial kernels due to its greater accuracy in terms of localized and finite responses. We ran the SVR from the *svm* package and tuned the parameter *C*, *epsilon*, and *gamma* through the *GridSearchCV* function from the *sklearn* package in Python 3.8.


(5) $$\min\;\frac{1}{2}\omega^{2}\;s.t.\;\;y_{i}-\left(\omega^{T}x_{i}+b\right)\leq \epsilon ,\;i=1,\;2,\;\ldots ,\;l$$

where (*ω*, *b*) is the hyperplane, (*x*_*i*_, *y*_*i*_)  is the sample point, *ϵ* is the tolerance deviation, and*l* is the sample size.

RFR is a bagging ensemble learning method for model training and prediction that integrates numerous decision trees lying on a collection of random variables sampled independently; the trees are then aggregated to produce a forest. Each decision tree yields a prediction from the samples and features drawn, and by combining the results of all the trees and taking the average, the regression prediction for the whole forest is obtained. By calculating the arithmetic mean, RFR can produce accurate predictions without a high computational burden (Breiman, 2001). The RFR ran in the *ensemble* package and the parameters *n_estimators*, *max_depth*, and *max_features* were tuned through the *RandomizedSearchCV* function from the *sklearn.ensemble* package in Python 3.8.

**Figure 3:**
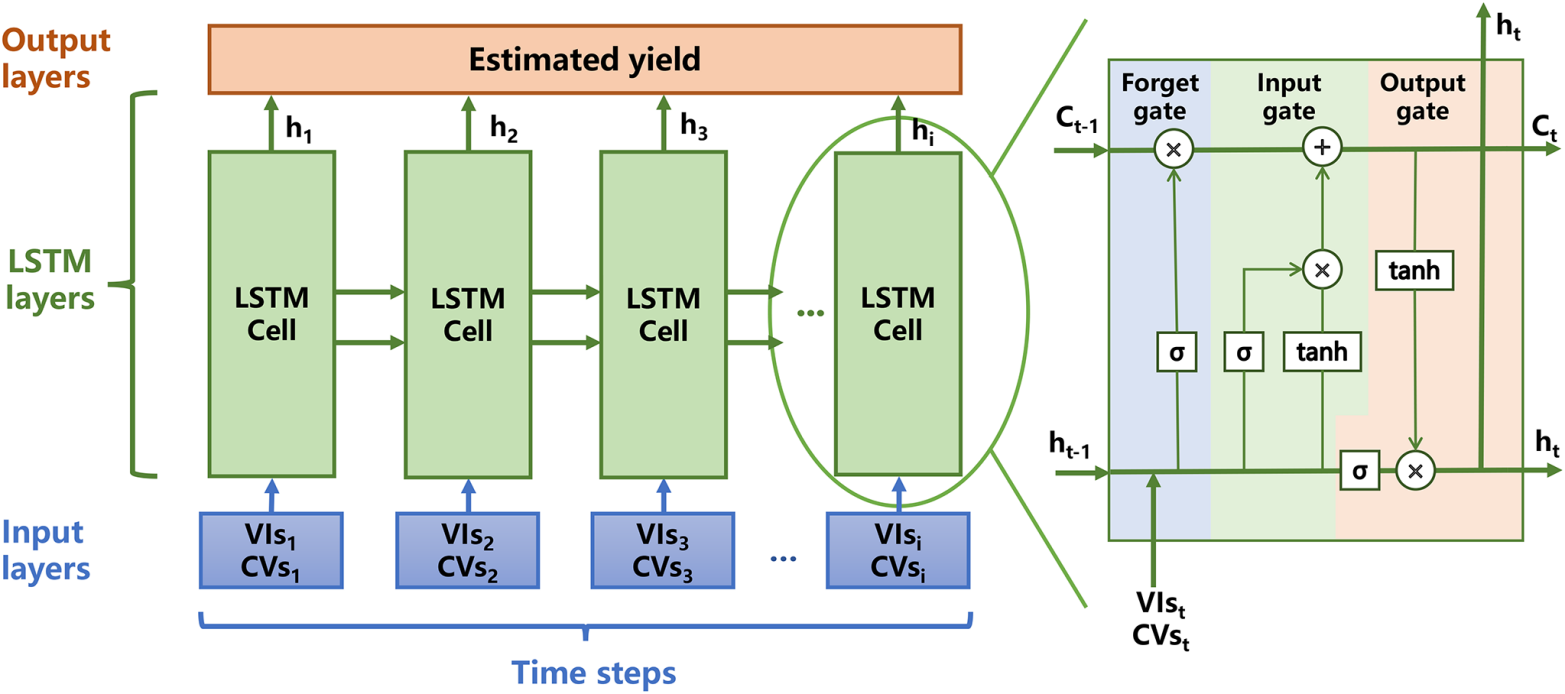
The architecture of long short-term memory (LSTM) model. The VIs variables refer to GI, RVI, NDVI. The CVs variables are soil, pet and vap.

LSTM is a special type of recurrent neural network (RNN) that can solve the problems of gradient disappearance and explosion and learn time-dependent information to understand crop growth processes (Hochreiter and Schmidhuber, 1997). These models include an input layer, one or more LSTM layers (consisting of LSTM cells), and an output layer. **Figure 3** shows the architecture of the LSTM model. Each LSTM cell contains forget, input, and output gates to determine which information to forget, retain, and output in the LSTM layers. Through the activation (σ) and tanh functions, the hidden neurons (h_t_) and internal memory cells (C_t_) renewed continuously, contributing to the memory ability of the network. We ran the LSTM model in MATLAB 2020, which contains the *lstmLayer* structure. The hyper-parameters were optimised by an *optimiseParameters* function that is created by ourselves to compare the accuracy of different parameter combinations and select the highest precision one. In this study, the networks were run for 60 epochs; the batch size was 10 in the learn rate drop period and the factors were 100 and 0.02. **Table 3** shows the specific parameters of the four models.

**Table 3:** The detail list of parameters used for the regression models.

Model	Parameters
LASSO	alpha = 0.1
SVR	C = 5000, gamma = 10, epsilon = 0.01
RFR	n_estimators = 120, max_depth = 12, max_features = 4
LSTM	miniBatchSize = 10, MaxEpochs = 60, LearnRateDropPreriod = 100, LearnRateDropFactor = 0.02

## Results

### Most important variables for estimating cotton yield

**Figure 4:**
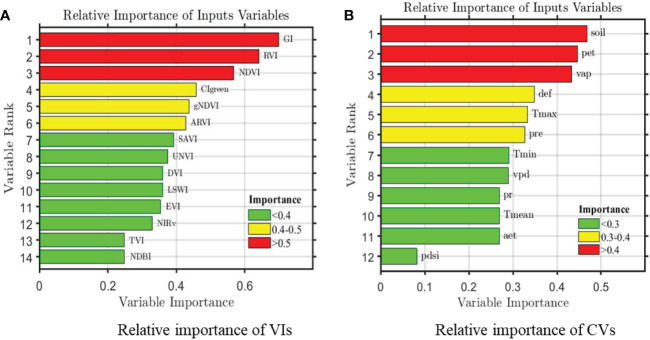
Relative importance of county-level remote sensing **(A)** and climate **(B)** variables on cotton yields during 2012-2019. Note: the aet, def, pdsi, pr, vap, vpd, pet, pre, soil, Tmax, Tmin, and Tmean represent actual evapotranspiration (mm), climate water deficit (mm), the palmer drought severity index, precipitation accumulation (mm), vapor pressure (kPa), vapor pressure deficit (kPa), reference evapotranspiration (mm), daily precipitation (mm), soil moisture (mm), monthly maximum, minimum and mean temperature (°C), respectively.

The ability of the 14 typical VIs listed in **Table 1** to predict cotton yield was evaluated using LASSO, SVR, RFR, and LSTM approaches. According to the relative importance of the variables, as illustrated in **Figure 4**, the green index (GI), ratio vegetation index (RVI), and NDVI contributed most to predictions of cotton yield in the study area, with importance values > 0.5; these were followed by CIgreen, gNDVI, and ARVI, with importance values of 0.4–0.5. The importance of the remaining variables did not exceed 0.4, indicating that cotton yield was little affected by them.

The three most important climate features for predicting yield were soil moisture, pet, and vap, with relative importance values of 0.47, 0.44, and 0.43, respectively. The other variables had importance values< 0.4. The least significant climate feature was pdsi, with an importance value< 0.1.

### Performance of satellite and climate data for cotton yield estimation

**Table 4** summarizes the yield estimation performance (mean values of 10-fold for training and testing results) achieved by applying the four regression algorithms using various parameters from 2012 to 2019. The important parameters were divided into VIs groups and VIs plus CVs groups. In experiments using both groups, the LSTM model outperformed the other models, followed by the two ML models (RFR and SVR). The linear regression model based on LASSO performed the worst, with non-linear relationships seen among the different predictors and cotton yield. Only the LSTM method had an R^2^ > 0.6, RMSE< 200 kg/ha and rRMSE< 11%. The two ML methods explained only 30-50% of the variance in cotton yield, with SVR performing slightly worse than RFR. We found that, with combined use of satellite and climate data as input variables, greater accuracy was achieved compared with the individual satellite data; R^2^ increased by 10%, and RMSE decreased by > 10 kg/ha, indicating that climate data provide complementary information that merits consideration. Our results suggest that the two datasets explain 66% and 76% of the cotton yield variability when using LSTM, respectively.

**Table 4:** The training and testing model performances (R^2^, RMSE and rRMSE in the average of 10-fold cross-validation) at county-level from 2012 to 2019 .

Model	Variables	Training R^2^	Training RMSE (kg/ha)	Training rRMSE	Testing R^2^	Testing RMSE (kg/ha)	Testing rRMSE
LASSO	VIs	0.25	223	13.94%	0.23	229	15.42%
	VIs+CVs	0.31	216	13.31%	0.27	207	13.74%
SVR	VIs	0.85	95	5.51%	0.35	224	12.93%
	VIs+CVs	0.95	85	4.94%	0.38	218	12.60%
RFR	VIs	0.85	83	5.03%	0.37	215	12.86%
	VIs+CVs	0.96	78	4.74%	0.47	208	12.50%
LSTM	VIs	0.98	65	3.60%	0.66	182	10.53%
	VIs+CVs	0.99	23	1.34%	0.76	150	8.67%

### Within-season predicting performance

**Figure 5:**
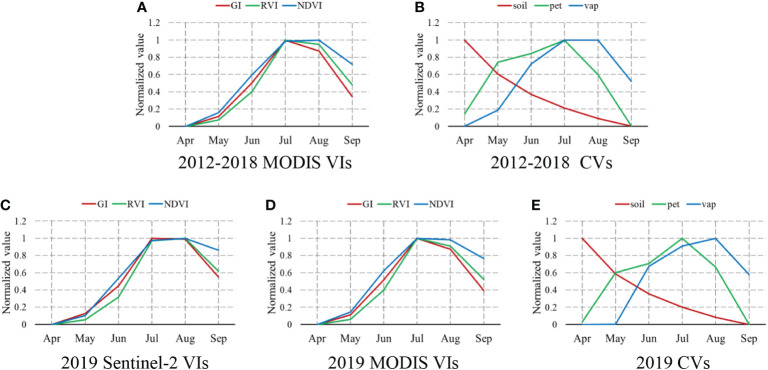
Normalized monthly means of the satellite (**A, C**, **D**) and climate (**B**, **E**) variables for the cotton study area for 2012-2018 (top row) and 2019 (bottom row), with raw values normalized to 0-1 to match their minimum and maximum values.

**Figure 6:**
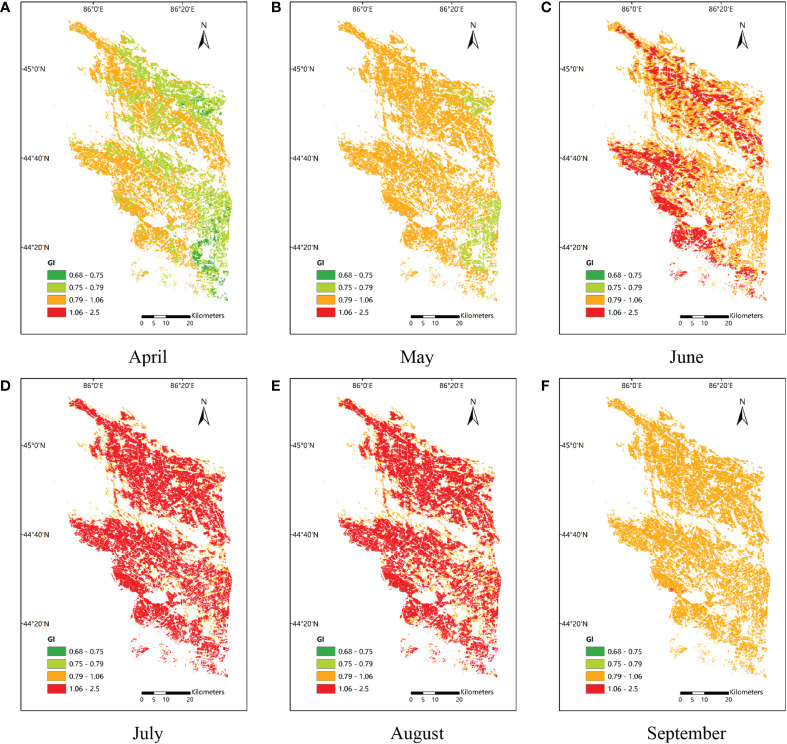
The spatiotemporal distributions of GI in Manas County in 2019, **(A–F)** refer to the different growth periods of cotton.

Using the most suitable variables and algorithms for predicting yearly yield, the seasonal cycles were examined (**Figure 5**). Generally, the values of all VIs (both MODIS and Sentinel-2 derived) increased gradually from April to July and the mid-summer peak during the cotton-blooming season (July–August), but peaked at different times between the two satellite systems. The three VIs peaked in July in the MODIS system, while in the Sentinel-2 system, only GI peaked in July and the seasonal cycles of RVI and NDVI lagged by 1 month. However, the difference between peak timings was very small. The satellite-derived VIs for July were very close to those for August. The GI and RVI derived from MODIS were clearly distinct from July to August, and dropped from 1 to 0.9. The NDVI derived from MODIS, and all three VIs derived from Sentinel-2, remained high, as in July. From August to September, the GI and NDVI declined the most and least rapidly, respectively. Since GI is the most important VIs, we explored the spatiotemporal pattern for Manas County (a large cotton growing area commonly used for research) in 2019 (**Figure 6**), verifying the reality of the whole county.

Of the CVs, pet and vap increased from April to July, peaking in July and August, respectively. The VIs had similar seasonal cycles, although pet decreased dramatically from the peak and reached its lowest value in September. The monthly variation in soil moisture had a different pattern from all other parameters examined. From the beginning of April to the end of September, it declined gradually from 1 to 0. There were no obvious differences among the green-up stage, blooming period, and cotton boll opening stage.

**Figure 7:**
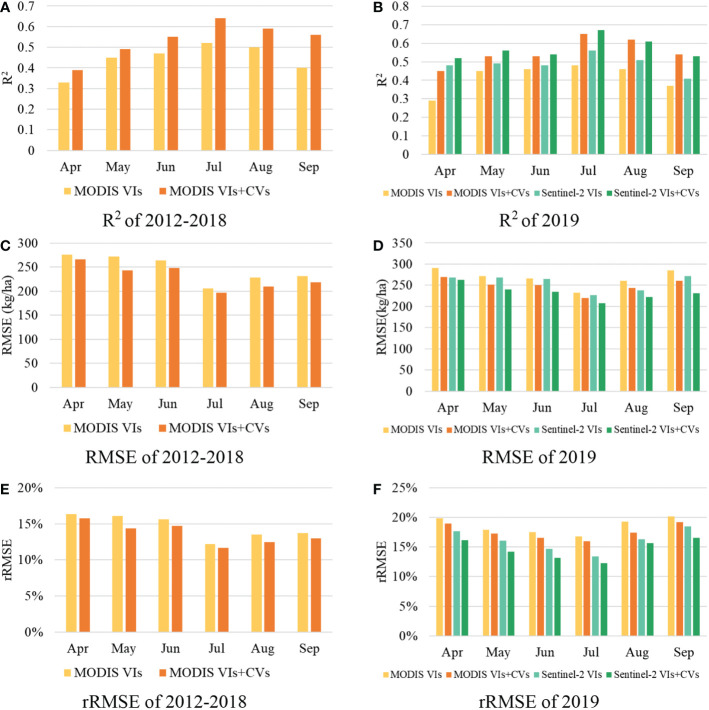
Testing performance [R2 **(A, B)**, RMSE **(C, D)** and rRMSE **(E, F)**] of cotton yield prediction only with remote sensing variables and combined with climate variables using the LSTM model for the whole growing season during 2012-2018 and 2019, respectively.

**Table 4** indicates that the LSTM model best predicted the yearly cotton yield at the county level. Hence, we used LSTM for the final regression model to analyze the within-season predicting performance for cotton in different months. To validate the MODIS satellite data, which has a spatial resolution of 500 m and may exceed the cotton field scale, we used Sentinel-2 data with a spatial resolution of 20 m to predict the yield in 2019, after training the model using data for 2012–2018. **Figure 7** shows the time series of the 10-fold averaged R^2^, RMSE and rRMSE, achieved with the LSTM method from April to September. The model showed poor performance during the early seedling and germination stages. As the cotton grew and developed, the information derived from the satellite data became more important. The estimation accuracy also increased gradually, peaking in July before starting to drop slightly in August. In September, when the cotton bolls began to open, the prediction accuracy decreased to a level close to that in June. The addition of CVs improved the ability of VIs to predict within-season production. From July to September R^2^ increased by 10%, RMSE and rRMSE decreased by 40 kg/ha and 3.25%, respectively; these values were much better compared with those for the green-up stage. Compared with the MODIS data, the Sentinel-2 data better predicted the cotton yield every month. After adding CVs to VIs as inputs, MODIS had essentially the same accuracy as Sentinel-2, revealing the feasibility of using MODIS data for county-level cotton yield prediction. Moreover, the 2019 validation experiment showed that MODIS can satisfactorily predict the cotton yield about 2 months before harvest (R^2^ = 0.65, RMSE = 220 kg/ha, rRMSE = 15.97% in July; R^2^ = 0.62, RMSE = 244 kg/ha, rRMSE = 17.39% in August). The Sentinel-2 data had slightly greater accuracy.

## Discussion

### The most suitable parameters for estimating Xinjiang cotton yield

Our first experiment examined which satellite data and CVs are most important for cotton yield estimation. After screening 14 VIs and 12 CVs, 3 of each showed clear superiority over the other parameters. The VIs GI, RVI and NDVI that with importance values > 0.5, and the CVs soil moisture, pet, and vap that with importance values > 0.4, performed best. They are significantly more important than the later ones. Like most plants, the reflectance for cotton is highest in the near-infrared band, with relatively less reflectance seen in the green band and an absorption valley occurring in the red band. VIs are an efficient way to measure crop growth and, ultimately, production (Meng et al., 2017; Ren et al., 2018). GI is defined as the ratio of the green and red bands. It is mainly influenced by the canopy chlorophyll concentration, and best explained the variability in cotton yield in this study. RVI is the ratio of the near-infrared and red bands. It is affected by vegetation structure and canopy nitrogen content, and is sensitive to atmospheric correction of the red band. Previous studies showed that NDVI is effective for estimating maize, rice, and soybean yield (Lambert et al., 2018; Cao et al., 2021). However, it often reaches a saturation point and is sensitive to the soil background, which may explain why it did not outperform GI and RVI in this study. Among the VIs with importance values exceeding 0.5, the red band was the most important. The first five VIs utilized only the information in the green, red, and near-infrared bands, illustrating their utility for estimating cotton yield. It’s due to the presence of chlorophyll, green plants strongly absorb radiation energy in the red band (> 90%) and form a green reflective peak in the green band (10% - 20%). Therefore, we think the importance of chlorophyll in cotton growth can’t be ignored. On adding the blue and short-wave infrared bands, the effects of the other VIs decreased gradually. DVI and TVI were unable to eliminate sensor and atmospheric effects. NIRv is multiplied by the near-infrared band and NDVI, and has been successfully applied for crop monitoring; however, it may eliminate certain types of canopy structure information (Zeng et al., 2022). NIRv did not estimate cotton yield well, suggesting that structure information cannot be ignored when making yield predictions. Overall, the VIs chosen herein to predict the Xinjiang cotton yield were characterized by high correlations with crop growth conditions in the canopy structure and the chlorophyll contents.

Among the CVs, soil moisture, pet, and vap best reflected the yield variation according to environmental factors. All three of these CVs are related to water, demonstrating that moisture greatly affects cotton yield. This is in line with the growth characteristics of cotton. In a field survey, we observed that cotton farmers used drip irrigation to overcome water shortages caused by insufficient rainfall. For most crops, precipitation and temperature are vital for yield prediction. However, the three CVs that we selected showed that precipitation was slightly more important than temperature. Based on a literature review, this discrepancy has two antecedents. First, the geographical vastness of our study area and great differences in altitude and terrain complexity lead to uneven rainfall and large differences in temperature, pressure, and soil type. Second, unlike precipitation and temperature, which are single indicators, soil moisture, pet, and vap are composite variables calculated from the former two variables. For growth, cotton must absorb water from soil and breathe *via* leaf evapotranspiration. Therefore, combining CVs with conventional satellite RS data can provide complementary information, thereby improving the accuracy of cotton yield estimation.

### Potential of the LSTM network for yield prediction

The results showed that the four statistical approaches performed differently. The two ML methods (SVR and RFR) and DL method (LSTM) performed better than the linear regression model (LASSO), consistent with previous studies (Gopal and Bhargavi, 2019; Zhang et al., 2021; Jeong et al., 2022). The reason for this may be that the LASSO algorithm lacks the ability to identify potential nonlinear and complicated relationships among input variables, which is the main strength of the other three models. The two ML methods exhibited average performance; SVR could not capture the relationship between yield and the other variables as well as RFR. We attributed this to an algorithm difference; RFR has excellent robust generalization ability, while SVR is limited by the quadratic programming problem. The limited sample size could be another reason why the ML models did not perform as well as expected, although we used 10-fold cross validation. LSTM can efficiently and effectively extract key temporal features hidden within input variables without the need for thousands of samples. Due to its RNN structure, LSTM is a useful DL approach for predicting crop yield. Furthermore, other studies have shown that RS data and climate features can reveal the complex reasons for yield variation (Cai et al., 2019; Kim et al., 2019; Cao et al., 2021). Therefore, we integrated satellite RS data and CVs to predict cotton yield at the county level. Compared with satellite VIs alone, all models performed better after the addition of CVs. This suggests that environmental data supplies additional information that RS data are unable to provide, and verified the effectiveness of combining the two types of data for cotton yield estimation. On the other hand, it seems that our results have worse performances than other studies (Ashapure et al., 2020; Jeong et al., 2022). Meanwhile, some yield prediction performances are even worse than ours (Zhang et al., 2021; Li et al., 2022b). Through comprehensive analysis, we attribute the reasons for poor results to the following two parts. The first one is the study scale. Compared with the county level, the pixel or field scale that can capture more details without the influence of complex background is much more elaborate. The second is the kinds of data sources. Rather than MODIS satellite data only, the climate data, soil property, geography, and topography can provide extra information. The more types of data sources, the higher the accuracy of yield estimation (Zhang et al., 2020; Cheng et al., 2022; Li et al., 2022b). However, apart from satellite and climate data, we have no access to other data for our study region, resulting in relatively poor results. All in all, our results demonstrate the advantage of integrating satellite and climate data for the prediction of cotton yield.

Finally, since LSTM outperformed all of the other algorithms, we explored how early it can predict the cotton yield and validated this using Sentinel-2 satellite data for 2019. Exploring the within-season performance of the selected variables, we also found phenological changes in Xinjiang cotton. After planting cotton seeds in April, we could predict cotton yield increasingly accurately until July, with the accuracy then decreasing in August and September. In rice and wheat crops, prediction accuracy is stable from July to harvest. Why does this discrepancy arise? Regarding the monthly changes of the selected VIs shown in **Figure 5**, we found the same trend as for the cotton estimation accuracy, which is in accordance with the process of cotton growth, but not that of rice and wheat. As cotton grows, the leaves become thicker and less soil is exposed from April to July. The bolls begin to open in August, which affects the satellite VIs directly; these start to decrease in August, thereby reducing the connection between the green VIs and yield. Furthermore, given the possibility of errors accumulating due to the low spatial resolution of the MODIS sensors, we also used Sentinel-2 data to estimate the cotton yield. The patterns were similar in both cases, demonstrating that MODIS satellite data can predict county-level yield accurately. The three CVs also varied with cotton growth, with pet and vap changing like the VIs, while soil moisture progressively decreased. Overall, the cotton yield estimate was most accurate 2 months before harvest. The accuracy of county-level cotton yield estimates did not increase with time after planting, although many factors influence the development and production of cotton in the full growth stage. Early yield estimation plays an important role in precision agriculture. It can assist farmers with field management before harvest, thus helping them to avoid further losses, and also helps the Department of Agriculture make marketing decisions pertaining to foods to maintain economic balance.

### Uncertainties and prospects

This study found that a combination of satellite and climate data can estimate cotton yield at the county level more accurately through the application of different approaches using the GEE and python platforms. LSTM showed the best performance. We successfully predicted the cotton yield 2 months before harvest using the LSTM model. However, like many other studies, ours had a few uncertainties and limitations. First, our yield estimation did not include all counties in Xinjiang Province. Xinjiang consists not only of counties, but also of “construction crops”. Moreover, these regions sometimes intersect, which makes it difficult for the national statistical office to collect yield data by county. Hence, after removing invalid data, production data were available only for part of Xinjiang. Second, our cotton distribution areas remained static in the period 2012–2019, but in actuality they differed over time. The cotton crop map for 2020 was used for the entire study period, which probably led to errors when generating VIs and CVs (as the land use varied from year to year between cotton and other fields). Future studies should consider updating the cotton maps annually to reduce errors in cotton yield estimation. In addition, more data types should be considered to predict cotton yield, by making full use of complementary information (Clevers and vanLeeuwen, 1996; Guan et al., 2017; Franz et al., 2020; Zhang et al., 2020). We used common VIs and CVs, and did not consider other data types. Solar-induced chlorophyll fluorescence (SIF) and synthetic aperture radar (SAR) data can also contribute to yield estimation. SIF is good at capturing the photosynthetic activity of plants (Duveiller and Cescatti, 2016; Kang et al., 2022), while SAR microwave data can assess plant structure due to its multi-polarization, multi-perspective scattering characteristics (Setiyono et al., 2019; Wu et al., 2020). Furthermore, the specific attributes of bolls compared with other crops and the spatial distribution characteristics of small and scattered cotton fields in Xinjiang should be considered. The domain knowledge-aware deep networks that take into account the enormous importance of small categories may offer a new way to conquer this problem (Li et al., 2022a). Finally, the spatial resolution of our major datasets was insufficient to reduce most of the errors affecting county-level predictions of cotton yield at the local scale; the satellite SR data and CVs used are only available at low spatial resolution, and the mixed pixels cannot distinguish cotton from other features, which reduces the accuracy of cotton yield prediction (Hunt et al., 2019; Meng et al., 2019). In the future, we may combine satellite data from different sensors with a higher temporal and spatial resolution to better extract the unique traits of cotton and improve cotton yield estimation accuracy.

## Data Availability

The original contributions presented in the study are included in the article/supplementary material. Further inquiries can be directed to the corresponding author.

## Conclusions

In this study, we pre-processed satellite data and CVs on the GEE platform and then identified the most important variables for cotton yield prediction at the county level in Xinjiang, using one linear regression (LASSO) and two ML (SVR and RFR) models, and one DL model (LSTM), with different combinations of input variables. The results showed that LSTM performed best, with an R^2^ of 0.76, RMSE of 150 kg/ha and rRMSE of 8.67% after an average of 10 runs. The performance was better after integrating RS and climate features. We used the LSTM algorithm, with VIs and CVs incorporated, to monitor cotton cropland during its growth and development. Finally, the within-season yield prediction suggested that cotton yield could be predicted reasonably accurately in July, 2 months before harvest, with an R^2^ of 0.65, RMSE of 220 kg/ha and rRMSE of 15.97%. The model using high-spatial-resolution Sentinel-2 data performed slightly better than the coarse MODIS data for yield predictions for 2019. The MODIS and Sentinel-2 data had the same monthly prediction accuracy, indicating that MODIS satellite data can satisfactorily estimate cotton yield in advance, thus facilitating cotton management decisions. To remove redundant features, the Boruta algorithm was used to determine which VIs and CVs were most sensitive to the county-level cotton yield in Xinjiang; this identified three VIs and three CVs. The VIs GI, RVI, and NDVI contain green, red and near-infrared bands, indicating that information on cotton canopy structure and chlorophyll contents can be useful for yield estimation. Because cotton fields are scattered throughout Xinjiang, only parts of each county are used to grow cotton, so the problem of mixed pixels must be considered. The most important CVs in this study were soil moisture, pet, and vap, which reflect moisture. Overall, MODIS satellite data integrated with CVs based on the LSTM model were superior for county-level cotton yield prediction in Xinjiang. In the future, the VIs characterizing canopy structure and chlorophyll and CVs related to moisture can be further investigated for cotton growth, and the LSTM method can be widely applied in crop yield prediction over large areas.
